# High-density lipoprotein ameliorates palmitic acid-induced lipotoxicity and oxidative dysfunction in H9c2 cardiomyoblast cells via ROS suppression

**DOI:** 10.1186/s12986-019-0356-5

**Published:** 2019-05-28

**Authors:** Kuen-Ming Wu, Yuan-Man Hsu, Mei-Chin Ying, Fuu-Jen Tsai, Chang-Hai Tsai, Jing-Gung Chung, Jai-Sing Yang, Chih-Hsin Tang, Li-Yi Cheng, Po-Hua Su, Vijaya Padma Viswanadha, Wei-Wen Kuo, Chih-Yang Huang

**Affiliations:** 1 Department of chest medicine, Jen-Ai Hospital, Taichung, Taiwan; 2 Department of Biological Science and Technology, China Medical University, Taichung, Taiwan; 3 Department of Food Nutrition and Health Biotechnology, Asia University, Taichung City, Taiwan; 4 Department of Medical Research, China Medical University Hospital, China Medical University, Taichung City, Taiwan; 5 School of Chinese Medicine, College of Chinese Medicine, China Medical University, Taichung, 40402 Taiwan; 6 China Medical University Children’s Hospital, China Medical University, Taichung, Taiwan; 7 Department of Healthcare Administration, Asia University, Taichung, Taiwan; 8 Department of Medical Research, China Medical University Hospital, China Medical University, Taichung, Taiwan; 9 Department of Pharmacology, School of Medicine, China Medical University, Taichung, Taiwan; 10 Chinese Medicine Research Center, China Medical University, Taichung, Taiwan; 11 Graduate Institute of Basic Medical Science, China Medical University, Taichung, Taiwan; 12 Department of Radiology, Jen-Ai Hospital, Taichung, Taiwan; 13 Department of Biotechnology, Bharathiar University, Coimbatore, 641 046 India; 14 Department of Biotechnology, Asia University, Taichung, Taiwan; 15 College of Medicine, Hualien Tzu Chi Hospital, Buddhist Tzu Chi Medical Foundation, Tzu Chi University, Hualien, Taiwan

**Keywords:** High-density lipoprotein, Palmitic acid, Lipotoxicity, Cardiomyoblast, ROS

## Abstract

**Background:**

High levels circulating saturated fatty acids are associated with diabetes, obesity and hyperlipidemia. In heart, the accumulation of saturated fatty acids has been determined to play a role in the development of heart failure and diabetic cardiomyopathy. High-density lipoprotein (HDL) has been reported to possess key atheroprotective biological properties, including cellular cholesterol efflux capacity, anti-oxidative and anti-inflammatory activities. However, the underlying mechanisms are still largely unknown. Therefore, the aim of the present study is to test whether HDL could protect palmitic acid (PA)-induced cardiomyocyte injury and explore the possible mechanisms.

**Results:**

H9c2 cells were pretreated with HDL (50–100 μg/ml) for 2 h followed by PA (0.5 mM) for indicated time period. Our results showed that HDL inhibited PA-induced cell death in a dose-dependent manner. Moreover, HDL rescued PA-induced ROS generation and the phosphorylation of JNK which in turn activated NF-κB-mediated inflammatory proteins expressions. We also found that PA impaired the balance of BCL_2_ family proteins, destabilized mitochondrial membrane potential, and triggered subsequent cytochrome c release into the cytosol and activation of caspase 3. These detrimental effects were ameliorated by HDL treatment.

**Conclusion:**

PA-induced ROS accumulation and results in cardiomyocyte apoptosis and inflammation. However, HDL attenuated PA-induced lipotoxicity and oxidative dysfunction via ROS suppression. These results may provide insight into a possible molecular mechanism underlying HDL suppression of the free fatty acid-induced cardiomyocyte apoptosis.

## Introduction

Atherosclerosis is considered to be a form of chronic inflammation and a disorder of lipid metabolism [1], elevated levels of serum cholesterol, low levels of HDL, diabetes mellitus, metabolic syndrome, are probably unique in being sufficient to drive the development of atherosclerosis in human and experimental animals, even in the absence of other known risk factors [2]. In heart, the accumulation of saturated fatty acids has been proposed to play a role in the development of heart failure and diabetic cardiomyopathy [3–5] as well as ischemia–reperfusion [5].

Palmitic acid, a 16-carbon saturated fatty acid (CH_3_ (CH_2_)_14_COOH), found in animals and plants, which is a major circulating saturated fatty acid. Excessive palmitic acid has been implicated in the induction of apoptosis in a large variety of cell types including cardiomyocytes [6–8]. The World Health Organization claims there is convincing evidence that dietary intake of palmitic acid increases risk of developing cardiovascular diseases (World Health Organization, Geneva, 2003, p. 88). Excess lipid may be stored as triglycerides, but are also shunted into non-oxidative pathways that disrupt normal cellular signaling leading to organ dysfunction and in some cases apoptosis, a process termed lipotoxicity [9]. Evidence is elevated levels of serum free fatty acid (FA) levels contribute to the pathogenesis of the metabolic syndrome and heart disease [10]. In heart, the accumulation of saturated fatty acids has been proposed to play a role in the development of heart failure and diabetic cardiomyopathy as well as ischemia–reperfusion [3, 4]. There are multiple pathways can be involved in the acute and chronic cellular effects of NEFA (non-esterified fatty acid) excess, such as reactive oxygen species production, mitochondrial permeability transition pore opening, IκB kinase and NF-κB activation, finally leading to cell dysfunction, apoptosis or necrosis [11]. ROS are involved in inflammation, endothelial dysfunction, cell proliferation, migration and activation, extracellular matrix deposition, fibrosis, angiogenesis, and cardiovascular remodeling, important processes contributing to cardiovascular and renal remodeling in hypertension, atherosclerosis, diabetes, cardiac failure, and myocardial ischemia-reperfusion injury [12, 13]. Oxidative stress induced by free fatty acids (FFAs) plays a key role in the development of cardiovascular diseases in metabolic syndrome [14]. Elevated FFA can cause oxidative stress due to increased mitochondrial uncoupling [15, 16] and β-oxidation [17, 18], leading to the increased production of ROS and the activation of stress-sensitive signaling pathways.

HDL are a fraction of serum lipoproteins, which is a strong, independent, inverse predictor of coronary heart disease risk [19–22]. HDL promotes the mobilization and clearance of excess cholesterol via the series of reactions collectively termed “reverse cholesterol transport” [23]. Another mechanism cited is that HDL possesses such as antioxidant capabilities, anti-inflammatory, anti-thrombotic, and anti-apoptotic activity [24].

Therefore, the aim of this study was to explore the mechanisms underlying HDL protects against palmitic acid-induced oxidative stress in cardiomyocytes. We investigated the ROS-mediated NF-κB activation and subsequent inflammatory and apoptotic signaling pathways.

## Materials and methods

### Cell culture

H9c2 cell lines were obtained from American Type Culture Collection (ATCC), cultured in Dulbecco’s modified essential medium (DMEM) supplemented with 10% Cosmic CalfR serum (CCS), 2 mM glutamine, 100 units/ml penicillin, 100 μg/ml streptomycin, and 1 mM pyruvate in humidified air (5% CO_2_) at 37 °C. During the treatment, pretreated with HDL for 2 h and then stimulated with palmitic acid (PA) for 24 h. The specificity of the inhibit ROS and mitochondria complex I inhibitor by adding N-acetyl cysteine (NAC) (500 μM).

### Lipoprotein separation

Human plasma was obtained from the Taichung Blood Bank (Taichung, Taiwan) and HDL was isolated using sequential ultracentrifugation (=1.019–1.063 g/ml) in KBr solution containing 30 mM EDTA, stored at 4 °C in sterile, dark environment and used within 4 days as previously described. HDL was separated from EDTA and from diffusible low molecular mass compounds by gel filtration on PD-10 Sephadex G-25 Mgel (Pharmacia) in 0.01 mol/l phosphate-buffered saline (136.9 mmol/l NaCl, 2.68 mmol/l KCl, 4 mmol/l Na_2_HPO_4_, 1.76 mmol/l KH_2_PO_4_) at pH 7.4. Protein concentration was determined by Bradford Protein Assay.

### MTT assay

MTT, [3-(4,5-Dimethylthiazol-2-yl)-2,5-diphenyltetrazolium-bromide]. The H9c2 cells were inoculated into 24-well plate. After HDL and palmitic acid treatments, the medium was removed and MTT solution (0.5 mg/ml) was added to each well which containing cells, subsequently incubated the plate in a 5% CO_2_ incubator at 37 °C for 1 h. MTT solution was replaced by isopropanol to dissolve blue formazan crystals, and absorbance was measured at 570 nm by using a microplate reader [25].

### DAPI staining and TUNEL assay

After various treatments, H9c2 cells grown on 6 mm plate were fixed with 4% paraformaldehyde solution for 30 min at room temperature. After a rinse with PBS, cells were treated with permeation solution (0.1% Triton X-100 in 0.1% sodium citrate) for 2 min at 4 °C. Following wash with PBS, samples were first incubated with Terminal Deoxynucleotide Transferase-mediated dUTP Nick End Labeling (TUNEL) reagent containing terminal deoxynucleotidyl transferase and fluorescent isothiocyanate-dUTP. The cells were also stained with 1 μg/ml DAPI for 30 min to detect cell nucleus by UV light microscopic observations (blue). Samples were analyzed in a drop of PBS under a fluorescence and UV light microscope, respectively. Apoptotic cells were assessed by fluorescence microscope or in a flow cytometer.

### Reactive oxygen species and mitochondrial superoxide production

Intracellular ROS generation was monitored by flow cytometry using peroxide-sensitive fluorescent probe 2′, 7′-dichlorofluorescein diacetate (DCFH-DA, Molecular Probes), dihydroethidium (DHE) and MitoSOX™ as a probe for the presence of H2O2 or superoxide. DCFH-DA is converted by intracellular esterases to DCFH, which is oxidized into the highly fluorescent dichlorofluorescein (DCF) in the presence of a proper oxidant, and then analyzed by flow cytometry. Dihydroethidium (DHE), by virtue of its ability to freely permeate cell membranes is used extensively to monitor superoxide production. It had long been postulated that DHE upon reaction with superoxide anions forms a red fluorescent product (ethidium) which intercalates with DNA. DHE is perhaps the most specific and least problematic dye; as it detects essentially superoxide radicals, is retained well by cells, and may even tolerate mild fixation. MitoSOX™ Red mitochondrial superoxide indicator is a novel fluorogenic dye for highly selective detection of superoxide in the mitochondria of live cells, which is rapidly and selectively targeted to the mitochondria. Once in the mitochondria, MitoSOX™ Red reagent is oxidized by superoxide and exhibits red fluorescence. MitoSOX™ is readily oxidized by superoxide but not by other ROS- or reactive nitrogen species (RNS)–generating systems, and oxidation of the probe is prevented by superoxide dismutase. The oxidation product becomes highly fluorescent upon binding to nucleic acids.

### Immunoblotting

Culture H9c2 cells were scraped and washed once with PBS, then cell suspension was spun down, and lysed in RIPA buffer (HEPES 20 mM, MgCl2 1.5 mM, EDTA 2 mM, EGTA 5 mM, dithiothreitol 0.1 mM, phenylmethylsulfonyl fluoride 0.1 mM, pH 7.5), and spun down 12,000 rpm for 20 min, the supernatant was collected in new eppendorf tube. Proteins (30 μg) were separated by electrophoresis on SDS-polyacrylamide gel. After the protein had been transferred to polyvinylidene difluoride membrane, the blots was incubated with blocking buffer (1X PBS and 5% nonfat dry milk) for 1 h at room temperature and then probed with primary antibodies (1:1000 dilutions) overnight at 4 °C, followed by incubation with horseradish peroxidase-conjugated secondary antibody (1:5000) for 1 h. To control equal loading of total protein in all lanes, blots were stained with mouse anti-β-actin antibody at a 1:50000 dilution. The bound immunoproteins were detected by an ECL kit.

### Measurement of mitochondria membrane potential

The lipophilic cationic probe fluorochrome 5,5′,6,6′-tetrachloro1,1′,3,3′-tetraethylbenzimidazolocarbocyanine iodide (JC-1) was used to explore the effect HDL on the mitochondria membrane potential (△Ψm). JC-1 exists either as a green fluorescent monomer at depolarized membrane potential or as a red fluorescent J-aggregate at hyperpolarized membrane potential. JC-1 exhibits potential-dependent accumulation in mitochondria, as indicated by the fluorescence emission shift from 530 to 590 nm. After treating cell with palmitic acid (0.5 mM) for 24 h in the presence or absence various concentrations of HDL, cell (5X104 cell/24-well plates) were rinsed with DMEM, and JC-1 (5 μM) was loaded. After 20 min of incubation at 37 °C, cell were examined under a fluorescent microscope. Determination of the △Ψm was carried out using a FACScan flow cytometer [26].

### Isolation of cytosolic fraction for cytochrome c analysis

After treating cells with palmatic acud in the presence and absence of natural products, the cells were collected and lysed with lysis buffer (20 mmol/L HEPES/ NaOH, pH 7.5, 250 mmol/L sucrose, 10 mmol/L KCl, 1.5 mmol/L MgCl2, 2 mmol/L EDTA, 5 mmol/L EGTA, 1 mmol/L DTT, protease inhibitor cocktail) for 20 min on ice. The samples were homogenized 30 strockes by glass Dounce and pestle. The homogenates were then centrifuged at 500x g to remove unbroken cells and nuclei. Supernatant were centrifuged at 17000x g for 30 min to isolate mitochondria fraction. Supernatant was cytoslic extraction and pellet was mitochondria fraction lysed by RIPA buffer. Cytosol and mitochondria protein were resolved by SDS-polyacryamide gel electrophoresis.

### Nuclear protein extraction

Cells grown to 80% confluency and subjected to various treatments were subsequently washed with ice-cold PBS and it was prepared for nuclear protein extraction. Cells grown on 10-cm dish were gently scraped with 3 ml ice-cold PBS and it were centrifuged at 1000x g for 10 min at 4 °C. After carefully aspirating the supernatant, cells were resuspended with 200 μl ice-cold BUFFER-I (10 mM Hepes (pH 8.0), 1.5 mM MgCl2, 10 mM KCl, 1 mM dithiothreitol, and proteinase inhibitor cocktail and incubated for 15 min on ice to allow cells to swell, followed by adding 20 μl IGEPAL-CA630. After vigorously vortexing for 10 s and centrifuging at 16,000 g for 5 min at 4 °C, the supernatant (cytoplasmic fraction) were carefully aspirated and the pellet were resuspended with ice-cold BUFFER-II (20 mM Hepes (pH 8.0), 1.5 mM MgCl2, 25% glycerol, 420 mM NaCl, 0.2 mM EDTA, 1 mM dithiothreitol and proteinase inhibitor cocktail and vigorously vortex. After vortexing, the suspension was placed on ice for 30 min before centrifuging at 16,000x g for 15 min at 4 °C. The supernatants (nuclear extracts) were stored aliquots at − 80 °C. Protein concentration of the supernatants was determined by the colorimetric assay.

### Transfection luciferase or siRNA assay

Transient transfections were carried out by the proprietary cationic polymer reagent (Fermentas) (TurboFect™ in vitro Transfection Reagent) following the manufacturer’s instruction. In some experiments 2 × 104 cells were plated onto 24-well plates and grown overnight. Vectors, including the reporter vectors, and the internal Renilla luciferase control vector (0.1 μg), and other protein expression vectors were cotransfected as indicated in the figure legends. All assays for firefly and Renilla luciferase activity were performed using one reaction plate sequentially. Briefly, at 24 h post-transfection and stimulation, the cells were washed with phosphatebuffered saline and lysed with Passive Lysis Buffer. After a freeze/thaw cycle, samples were mixed with Luciferase Assay Reagent II, and the firefly luminescence was measured with a Luminometer. Next, samples were mixed with the Stop & Glo reagent, and the Renilla luciferase activity was measured as an internal control and to normalize the luciferase activity values. Double-stranded siRNA sequences targeting JNK, NF-κB mRNAs were obtained from Santa Cruz Biotechnology. The non-specific siRNA (scramble) consisted of a nontargeting. Cells were cultured in 60-mm well plates in medium. Transfection of siRNA was carried out with transfection reagent. Specific silencing was confirmed by immunoblotting with cellular extracts after transfection.

### Annexin V-FITC/PI staining

H9c2 cells seeded at a density of 2 × 105 cells/well in 6-well plates were exposed to hypoxia for 24 h. Apoptotic cells were monitored by FACSCanto flow cytometry using the Annexin V-FITC Apoptosis Detection Kit. Total cells and supernatants were collected, washed and incubated for 15 min with 1 × binding buffer containing annexin V-conjugated fluorescein isothiocyanate (FITC) and propidium iodide (PI). Annexin V positive cells were considered as early apoptotic cells. Cells with annexin V and PI positive were considered as late apoptotic and/or necrotic cells whereas viable cells were unstained.

### Cardiomyocyte culture

Neonatal cardiomyocytes were isolated and cultured using the commercial Neonatal Cardiomyocyte Isolation System Kit according to manufacturer’s directions. Briefly, hearts from one- to two-day-old Sprague-Dawley rats were removed, the ventricles were pooled, and the ventricular cells were dispersed by digestion solution at 37 °C. Ventricular cardiomyocytes were isolated and cultured in DMEM containing 10% fetal bovine serum, 100 μg/ml penicillin, 100 μg/ml streptomycin, and 2 mM glutamine. After 3–4 days, cells were incubated in serum-free essential medium overnight before treatment with indicated agents.

### Statistical analysis

Statistical differences were assessed by one way-ANOVA. *P* < 0.05 was considered statistically significant. Data were expressed as the mean ± SEM.

## Results

### Palmitic acid (PA)-induced apoptosis, and cells death

**Fig. 1:**
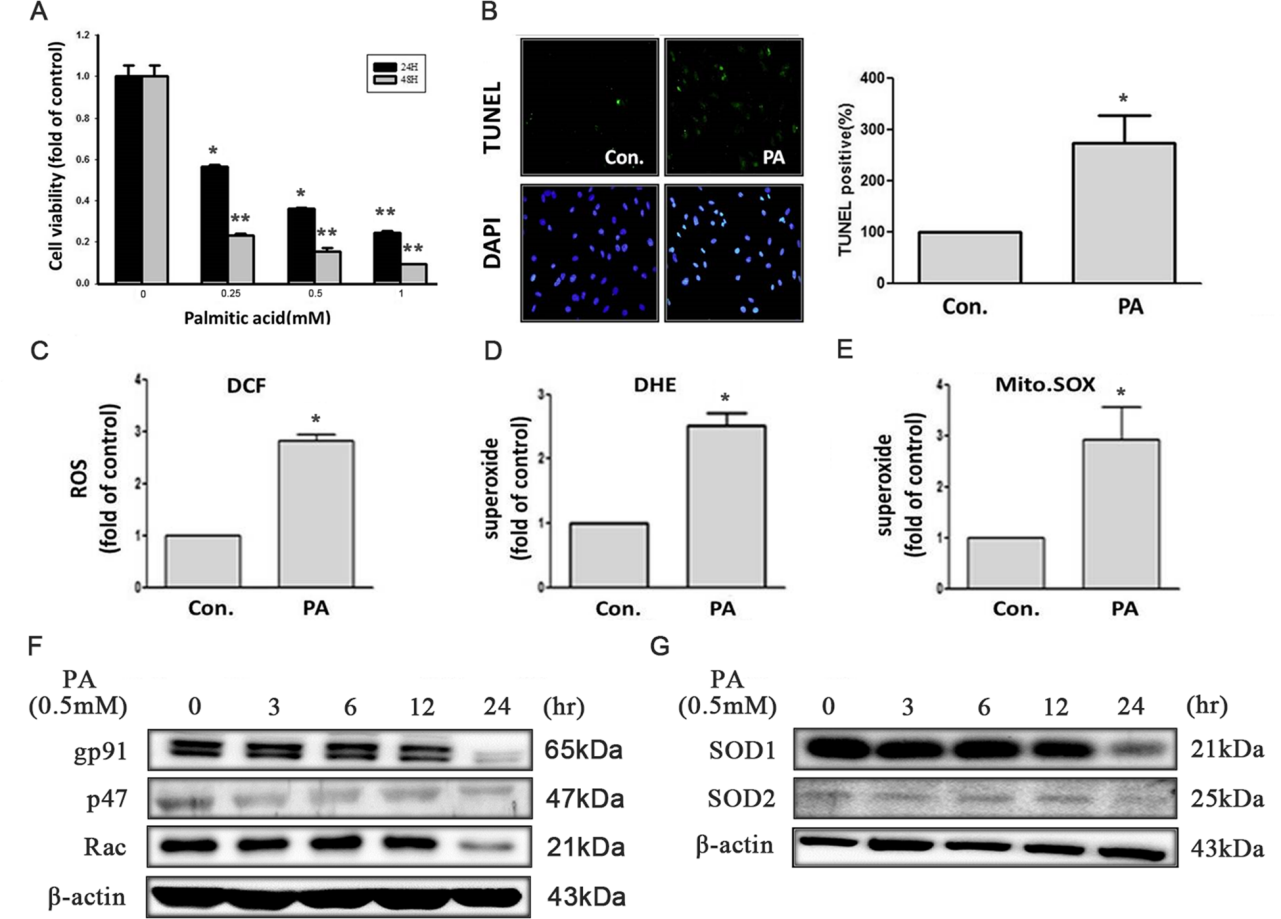
PA increased oxidative stress and induced cell death in H9c2 cells. **a** H9c2 cells were treated with PA at different concentrations for 24 or 48 h. Cells viability was measured by MTT assay. **b** H9c2 cells were incubated with PA (0.5 mM) for 24 h. Cells were stained with 4,6-diamidino-2-phenylindole (DAPI) and terminal deoxynucleotidyl transferase dUTP-mediated nick-end labeling (TUNEL) assay. H9c2 cells were treated with PA (0.5 mM) for 24 h followed by 1 h incubation with fluorescent probe (**c**) DCF-AM (10 μM) (**d**) DHE (10 μM) (**e**) MitoSOX™ (5 μM). Fluorescence intensity of cells was measured by flow cytometry. H9c2 cells were treated with PA (0.5 mM) for indicated time. **f** The levels of NADPH oxidase (Nox2-gp91, p47phox, Rac) and (**g**) antioxidant enzymes (SOD1, SOD2) were measured by Western blot. Data showed the means ± SEM of 3 independent analyses.*p < 0.05 and **p < 0.01 compared with the control

To clarify palmitic acid induced cytotoxicity in cardiomyocyte, H9c2 were treated with different concentrations of PA for 24 and 48 h. The result of MTT showed that after treatment with various concentrations of PA for indicated time period significantly decreased the cell viability in a dose-dependent manner (Fig. 1a). The cell viability is lower than 50% in concentration of PA on 0.5 mM treated with H9c2 cells, therefore 0.5 mM was used for the following experiments. We also used TUNEL analysis for observing cells undergoing apoptosis. After incubation with PA for 24 h, we observed a significant increase in apoptotic cells (Fig. 1b).

### Palmitic acid increased generation of mitochondrial reactive oxygen species (ROS)

Previous investigation demonstrated that free fatty acid (FFA) induced-oxidative stress plays an important key role in development of cardiovascular disease in metabolic syndrome [14]. We therefore, examined the cellular ROS levels after treatment with 0.5 mM PA for 24 h by fluorometric assay using DCF-AM and DHE. As shown in Fig. 1c and d, an approximately three-fold and two-fold increase of ROS and superoxide was observed in cells incubated with PA compared with untreated cells. NADPH oxidase and mitochondrion are known major sources of superoxide [27], so we measured the expression levels of NADPH oxidase subunits by Western blot (Fig. 1f) and generation of superoxide in mitochondria by MitoSOX™ Red (Fig. 1e). In Fig. 1e, an approximately three-fold increase of mitochondrial superoxide was observed in cells incubated with 0.5 mM PA for 24 h compared with normal condition. However, the protein levels of gp91phox, p47phox, Rac-1 protein in H9c2 cells in a time dependent manner (0-24 h) (Fig. 1f).

Intracellular ROS levels are regulated by the balance between ROS generation and antioxidant enzymes such as catalase or SOD. Besides, the involved ROS are able to inactivate antioxidative enzymes that additionally increase the imbalance in favor of oxidative stress. Therefore, we investigated the expression of its isoforms in H9c2 cells in response to PA. Our results showed that the antioxidant enzymes SOD1 and SOD2 decreased in H9c2 cells treatment with PA for 0.5 mM (Fig. 1g).

### Palmitic acid led to collapse of mitochondria member potential

**Fig. 2:**
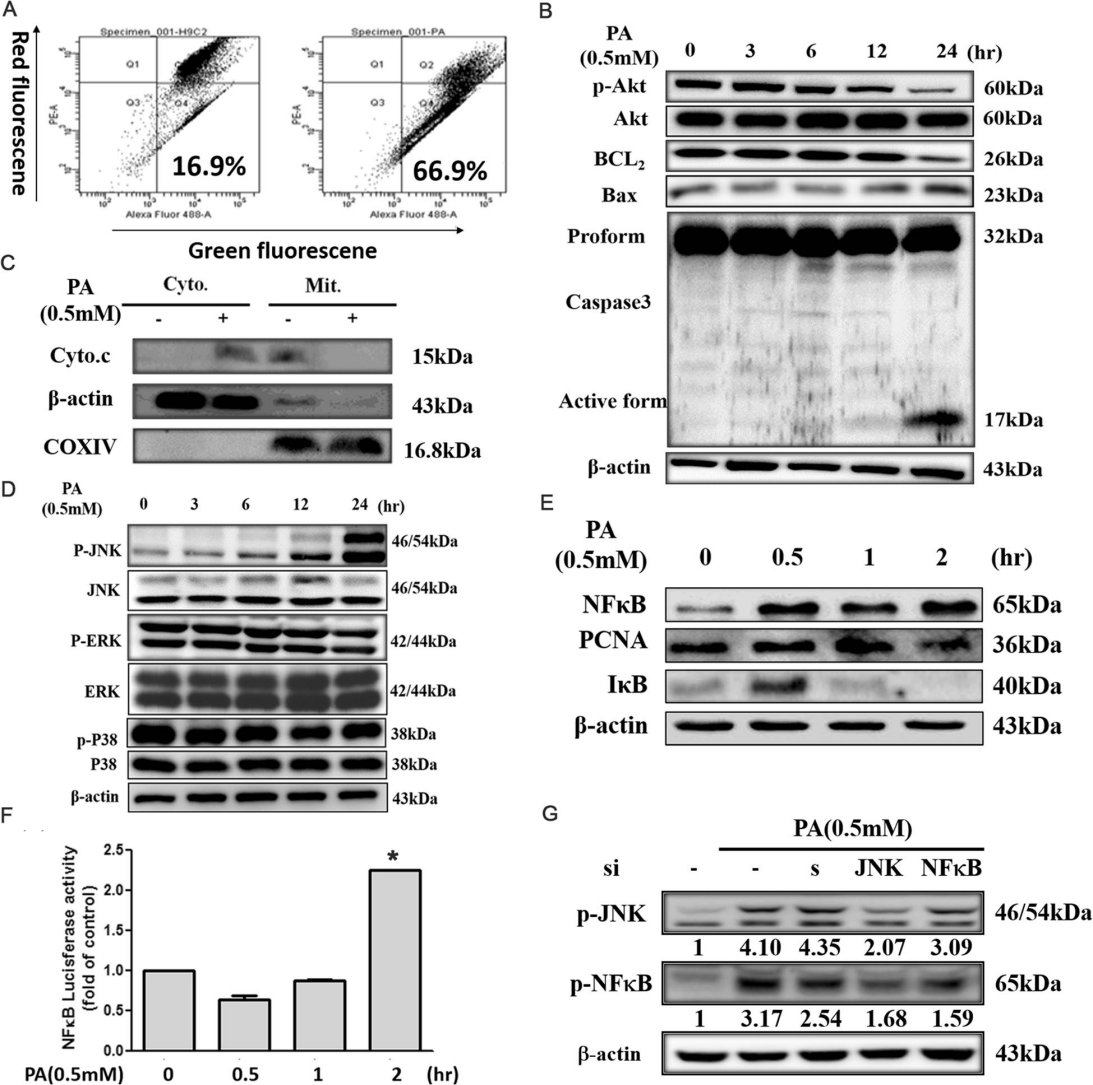
PA leads to unstability of mitochondria member potential triggered cell apoptosis through mitochondria dependent pathway and NFκB signaling pathway. **a** H9c2 cells were treated with PA (0.5 mM) for 24 h. △Ψm was assessed with signal from monomeric and J-aggregate JC-1 fluorescence. JC-1 fluorescence was measured by flow cytometry. Left: control, Right: PA. **b** H9c2 cells were treated with PA (0.5 mM) for indicated time. p-Akt, Bcl-2, Bax, caspase 3 expression was estimated by immunoblotting.(**c**) H9c2 cells were treated with PA (0.5 mM) for 24 h and the cell lysates were fractionated into cytosolic and mitochondrial proteins. Cytochrome c was analyzed by immunoblotting. β-actin and COX IV served as the cytosolic and mitochondrial loading controls. **d** H9c2 cells were treated with PA (0.5 mM) for indicated time. The expression of MAPK family (p-ERK, p-JNK, p-P38) was analyzed by immunoblotting. **e** H9c2 cells were incubated with PA (0.5 mM) for 0-2 h. The expression of NFκB, and IκB was analyzed by immunoblotting. β-actin and PCNA served as the cytosolic and nuclear loading controls. **f** Cells were transfected with a luciferase NFκB reporter construct. After transfection and treatment with PA for indicated time (0, 0.5, 1 or 2 h), the cells were assayed for luciferase activity. *p < 0.05 compared with the control. **g** After H9c2 cells were transfected with JNK1, NFκB siRNA (10 nM) for 24 h, followed by treatment of PA for 24 h, scramble for nonspecific siRNA control. The levels of proteins indicated were analyzed by Western blot

To examine whether influence of mitochondrial disruption accounts for the apoptosis effect of PA, we tested the effect of PA on mitochondrial permeability. When H9c2 cells were exposed to PA (0.5 mM), the △Ψm was depolarized, quantitative analysis from flow cytometry supported these findings (Fig. 2a).

### Palmitic acid induced-apoptosis involved in a mitochondrial- dependent pathway

Bcl2 family proteins are upstream regulators of mitochondrial membrane potential. Since PA depolarized △Ψm, whether PA also influenced Bcl2 family protein was investigated. After treated PA for indicated time (0–24 h), the immunoblotting studies demonstrated that PA downregulated the anti-apoptotic (Bcl2 and p-Akt^Ser473^) and upregulated the proapoptotic (Bax) proteins, also increased caspase 3 activity in H9c2 cells (Fig. 2b).

It is known that disruption of mitochondrial membrane function results in the discharge of the mitochondrial enzyme cytochrome c into the cytosol. Consequently, mitochondria were separated from the cytosolic fraction and detected by Western blotting. As show in Fig. 2c, the amount of cytochrome c released into the cytosolic fraction was much greater in H9c2 cells that had been incubated with PA for 24 h than in control cells. The results showed that PA significantly induced release of cytochrome c.

### Role of MAPK family proteins, NFκB signaling pathway in PA- induced apoptosis

To investigate whether MAPK family proteins were involved in the apoptosis-related signaling pathways activated in H9c2 treated with PA, we examined the expression levels of MAPK family proteins by Western blot. Our results showed that the phosphorylation of JNK, but not ERK or p38 was increased after treatment with PA for 24 h (Fig. 2d).

Accumulated evidence indicated that IκB kinase/NF-κB (IKK/NFκB) signaling pathways play critical roles in a variety of physiological and pathological processes many stimuli activate NF-κB, mostly through IκB kinase–dependent (IKK-dependent) phosphorylation and subsequent degradation of IκB proteins. The liberated NFκB dimers enter the nucleus, where they regulate transcription of diverse genes encoding cytokines, growth factors, cell adhesion molecules, and pro- and antiapoptotic proteins. In accordance with previous findings, our results showed protein level of NFκB was increasing in the nuclear fraction (Fig. 2e). In addition, cells were transfected with construct containing the NFκB-responsive luciferase reporter gene (NFκB Luc) to further confirm the effects of PA on NF-κB activation. The result of luciferase assay indicated that NFκB promoter activity was increasing in a time-dependent manner (Fig. 2f). In order to identify whether JNK is an upstream regulator of NF-κB, we knockdown JNK and NF-κB by si-RNA (10 nM) to advance study PA-induced apoptosis pathway. Cells were transfected with si-RNA for 24 h. The data showed NFκB si-RNA had no effect on PA-induced JNK activity (Fig. 2g). These observations indicate that JNK/NFκB pathway could mediate cardiomyocyte cell apoptosis induced by palmitic acid, but NFκB has no influence on JNK activity.

### HDL downregulated palmitic acid-induced apoptosis

HDL is a complex, bioactive particle, containing multiple acute phase response proteins, protease inhibitors, and complement regulatory proteins. So, I would like to know whether HDL can downregulated palmitic acid-induce apoptosis in H9c2 cardiomyocytes cells.

**Fig. 3:**
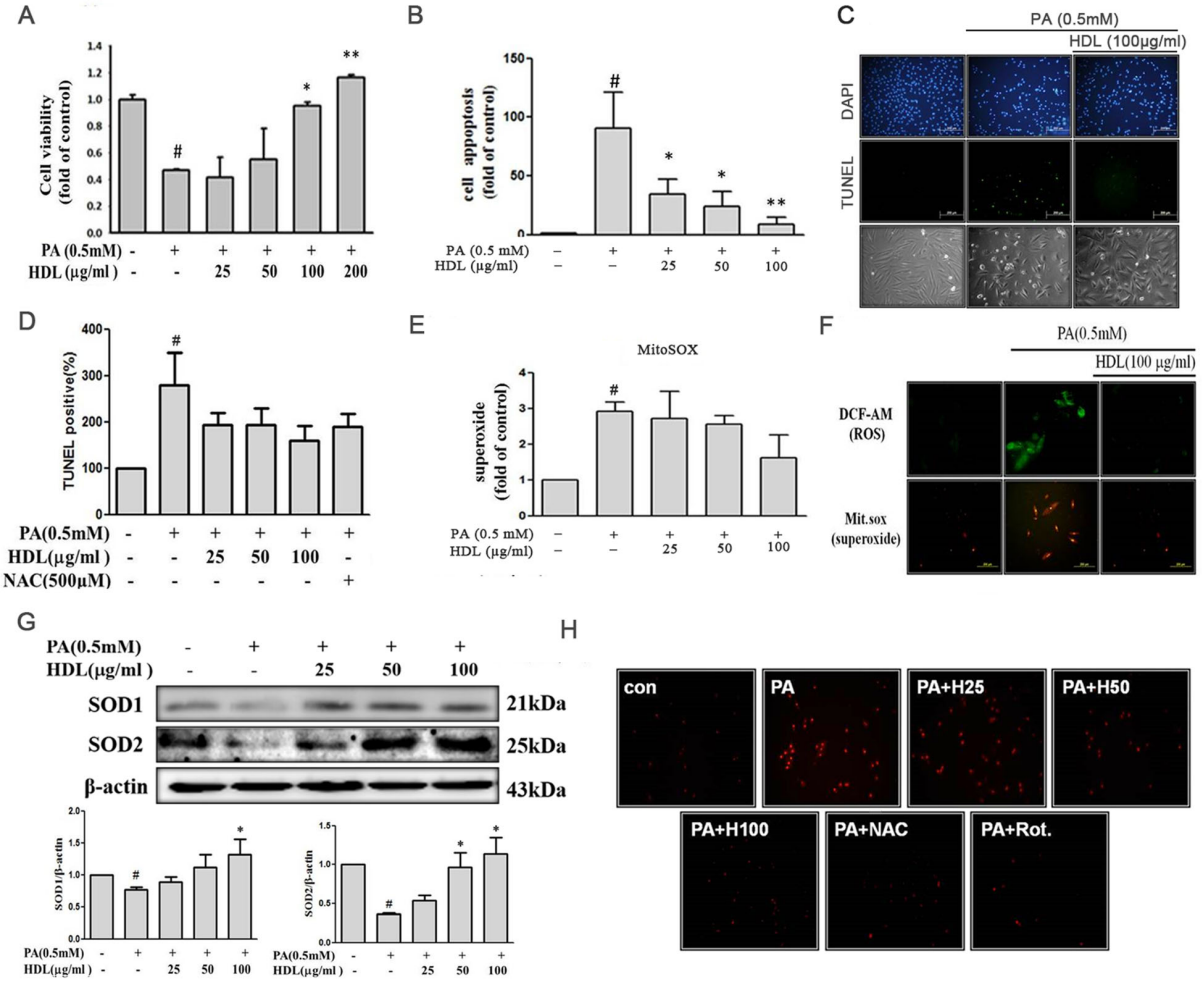
HDL attenuated PA-induced ROS production and cell apoptosis**.** H9c2 cells were incubated with PA (0.5 mM) in the absence or presence of different concentrations of HDL (25-100 μg/ml) for 24 h. **a** Cell viability was determined via MTT assay. **b** Flow cytometry profile represents Annexin-V-FITC staining in *x* axis and PI in *y* axis. The number represents the percentage of early apoptotic cells in each condition. **c** Fluorescence images showed the cells stained with 4,6-diamidino-2-phenylindole (DAPI) (upper panel) and stained using terminal deoxynucleotidyl transferase dUTP-mediated nick-end labeling (TUNEL) assay (middle panel), and photomicrographs were from phase-contrast microscopy (bottom panel). **d** TUNEL positive cell was determined via flow cytometric analysis. **e** Cellular ROS was determined via MitoSOX™ (5 μM). **f** Fluorescence intensity of cells was measured by phase-contrast microscopy. **g** SOD1 and SOD2 expression was estimated by immunoblotting. **h** Neonatal cardiomyocytes were treated with HDL 100 μg/ml for 2 h and then incubated with 0.5 mM PA for an additional 24 h, and followed by 1 h incubation with MitoSOX™ (5 μM). Fluorescence intensity of cells was measured by phase-contrast microscopy and. Data showed the means±SEM of 3 analyses. # *p* < 0.05 vs. control; *p < 0.05 and **p < 0.01 vs. palmitic acid alone treatment

The viability of cells incubated with PA in the absence or presence of indicated concentrations of HDL was assessed using the MTT assay (Fig. 3a). The result showed that PA significantly reduced viability in H9c2 cells after 24 h of incubation; however, pretreatment with HDL inhibited PA-induced cytotoxicity of H9c2 cells dose dependently. Next, we examined whether HDL possesses antiapoptotic effects in PA-treated H9c2 cells. To further determine whether HDL could against PA-induced apoptosis, PA-treated cells were analyzed biochemically via Annexin V binding assay (Fig. 3b) and TUNEL and DAPI staining assay (Fig. 3c) and evaluated by flow cytometry (Fig. 3d) and microscopic observation (Fig. 3c). Our results showed that the cells showed typical features of apoptosis, including the formation of compressed nuclei after treated with PA for 24 h, which were, however, not observed in the HDL-pretreated H9c2 cells and also reduced the phenomenon of apoptosis in dose dependently. Phase-contrast microscopy was performed to examine the protective effects of HDL on morphological features of H9c2 cells after exposure to PA, the number of shrunken cells of cells with blebbing membranes was significantly reduced by the presence of HDL (Fig. 3c) and anti-oxidant (NAC,500 μM) (Fig. 3d).

### HDL inhibits palmitic acid-induce ROS and superoxide generation

Studies demonstrated that PA elevated the concentration of cellular ROS, which subsequently led to the change the cell signaling pathway to mediate cell dysfunction. Therefore, we investigated the effects of HDL on generation of ROS, a potential factor related to PA-induced H9c2 cells injury, by hydroxyl radical sensitive probe 2′,7′-dichlorofluorescein acetoxymethyl ester (DCF-AM) (Fig. 3f) and MitoSOX™ Red mitochondrial superoxide indicator (Fig. 3e). The levels of ROS and mitochondrial superoxide generation significantly decreased in H9c2 cells after pretreatment with HDL (25-100 μg/ml) for 2 h before exposure to PA (0.5 mM). Intracellular ROS levels are regulated by the balance between ROS generation and antioxidant enzyme. Besides, the involved ROS are able to inactivate antioxidative enzymes that additionally increase the imbalance in favor of oxidative stress. Therefore, we clarify the expression of SOD isoforms in H9c2 cells in response to PA. The results showed that HDL significantly reduced the suppression of SOD activity caused by PA, the levels of SOD1 and SOD2 was decreased after PA treatment for 24 h. However, pretreatment H9c2 cells with HDL (25-100 μg/ml) significantly rescued the levels of SOD1 and SOD2 expression (Fig. 3g).

### Sustain exposures HDL can reduce mitochondrial ROS in neonatal cardiomyocytes treated with PA

To verify lipotoxicity could induce cardiac cellular ROS generation, and HDL could attenuate this phenomenon. We examined the cellular of mitochondrial superoxide generation in cultured primary rat neonatal cardiomyocytes. Pretreatment of neonatal cardiomyocytes with HDL (25-100 μg/ml) for 2 h before exposure to PA for 24 h. We used MitoSOX™ Red mitochondrial superoxide indicator to confirm by microscopic observation (Fig. 3h). PA enhanced mitochondrial superoxide generation. However, pretreated with HDL (25-100 μg/ml) attenuated PA-induced upregulation of mitochondrial superoxide generation. Moreover, anti-oxidant (NAC,500 μM) and mitochondrial superoxide inhibitor (Rotenone,5 μM) treatment also reversed the effects induced by PA.

### HDL stabilized on mitochondrial transmembrane permeability transition

**Fig. 4:**
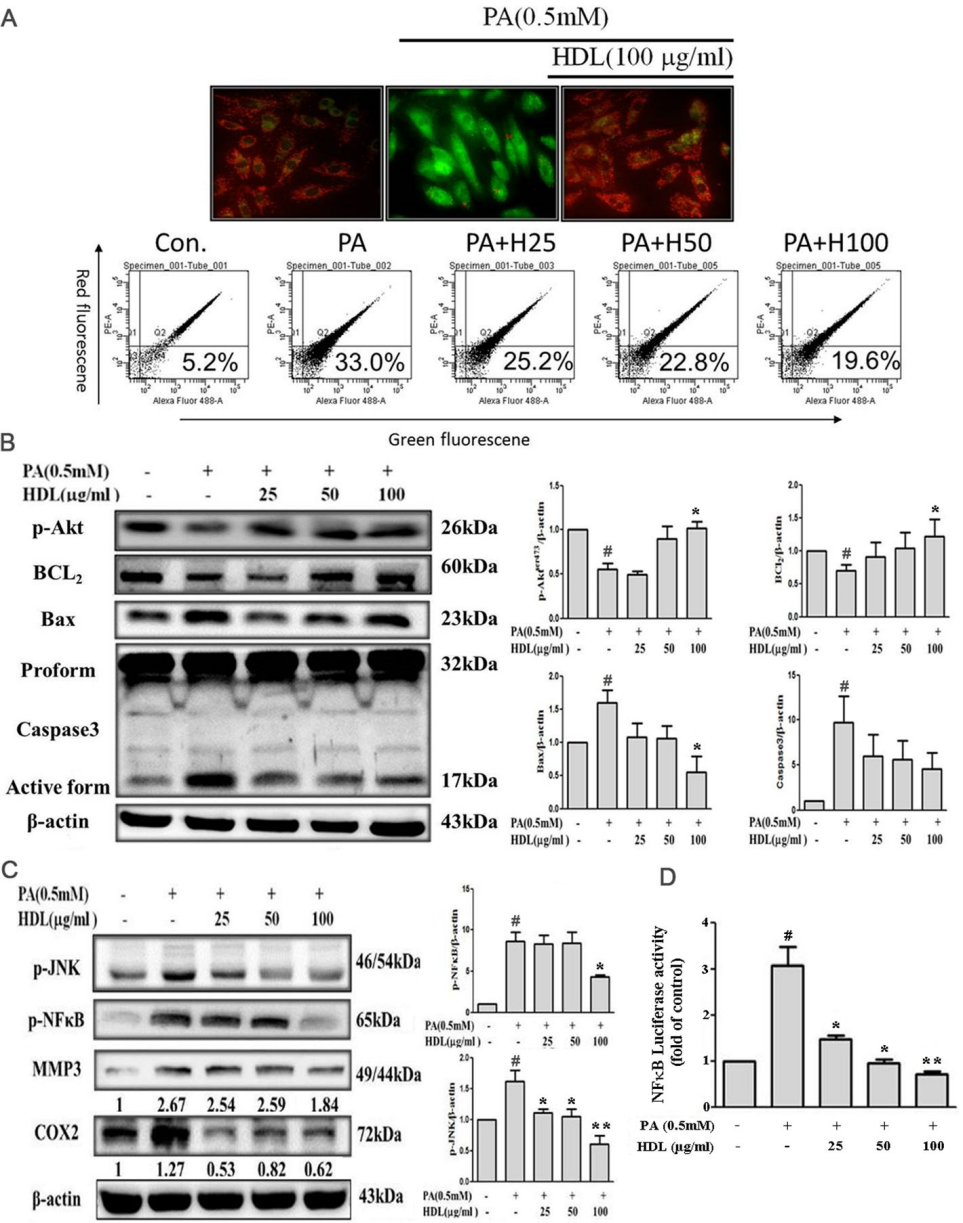
HDL stabilized on mitochondrial transmembrane permeability transition (△Ψm) and downregulated PA-triggered mitochondrial dependent pathway, JNK phosphorylation and NFκB activity in H9c2 cells. **a** Cells were incubated with HDL 100 μg/ml for 2 h and then incubated with 0.5 mM PA for an additional 24 h. The change in mitochondrial membrane poteneial was assessed based on the signal intensity from monomeric (green) and J-aggregate (red) JC-1 fluorescence. No treatment (left); PA (middle); and PA + HDL (right). **b** H9c2 cells were pretreated with the indicated concentrations of HDL (25-100 μg/ml) for 2 h followed by PA (0.5 mM) treatment for 24 h. p-Akt, Bcl_2,_ Bax, Caspase 3 expression was estimated by immunoblotting. **c** p-JNK, p-NFκB, MMP3, COX2 was estimated by immunoblotting. **d** Cells were transfected with a luciferase NFκB reporter construct. After transfection and treatment with PA and indicated concentrations of HDL (25-100 μg/ml), the cells were assayed for luciferase activity. Data showed the means±SEM of 3 independent analyses. # *p* < 0.05 vs. control; *p < 0.05 and **p < 0.01vs. palmitic acid alone treatment

To examine whether inhibition of mitochondrial disruption accounts for the anti-apoptotic effect of HDL, we examined the effects of HDL on mitochondrial permeability. When H9c2 cells were exposed to PA (0.5 mM), the △Ψm was depolarized, as shown by the increase in green fluorescence. Pretreatment with HDL reduced the change in △Ψm, as indicated by repression of green fluorescence and restoration of red fluorescence. As shown in Fig. 4a, PA caused a marked increase in JC-1 green fluorescence (middle) compared with the control (left). Pre-treatment with HDL (100 μg/ml) caused marked inhibition of PA-induced apoptotic (right).

### HDL restores survival protein expression and suppresses caspase 3 activity

Since PA depolarized △Ψm whereas HDL maintained it, whether HDL influenced the equilibrium of Bcl-2 family proteins was investigated. Immunoblotting studies demonstrated that PA downregulated the antiapoptotic and survival protein (Bcl2, p-Akt^Ser473^), also upregulated the proapoptotic protein (Bax), whereas HDL pretreatment effectively repressed these PA-induced proapoptotic events (Fig. 4b). Therefore, activated caspase 3 is a key factor in the execution of mitochondrial apoptosis [28]. Whether PA and HDL ultimately influence this factor to modulate apoptosis, we subsequently determined the pro-form and active-form of caspase 3 by immunoblotting (Fig. 4b). The data showed that active caspase 3 was significantly increased in PA alone group but active caspase 3 was suppressed in cells that had been pretreated with HDL.

### HDL decreased p-JNK and p-NFκB protein expression, and down-regulation of promoter activity in H9c2 cardiomyoblast cells

It has been shown that the transcriptional factor NFκB can be induced by a multitude of stimuli, including cytokines and ROS. However, in cardiomyocytes, NFκB activation has been found to produce cell apoptosis instead of preventing the cells from apoptosis. Therefore, we determined whether HDL inhibits NFκB-triggered downstream inflammatory proteins in H9c2 cells. We investigated the immunoblotting of p-NFκB, p-JNK and NFκB-triggered downstream inflammatory proteins, to determine whether HDL inhibits the phenomenon. As show in Fig. 4c, pretreated with HDL (25-100 μg/ml) significantly inhibited p-NFκB, p-JNK, COX2, and MMP-3 protein expression in a dose-dependent manner. Further, the activation of NFκB was measured in terms of its ability to promote of target gene expression. The result of the luciferase assay were used to represent the activity of NFκB regulate the expression of its target genes. Cells transiently transfected with NFκB luciferase plasmid after 24 h were exposed to PA with different concentrations of HDL (25-100 μg/ml) for 24 h. As shown in Fig. 4d, there was an approximately threefold increase in luciferase activity in H9c2 cells stimulated with PA as compared with control. Pretreatment with HDL (25-100 μg/ml) inhibited PA-induced luciferase activity in a dose-dependent manner. These finding indicated that PA causes activation of NFκB, and HDL could significantly inhibit NFκB activity.

## Discussion

In the present study we found that palmitic acid induced significant apoptosis and ROS generation in H9c2 at the concentrations above 0.5 mM. Although some papers were obtained palmitic acid with this ratio, it is different from the most often used 2/1 ratio [6, 29, 30].

Dietary fats modify the composition of cellular and mitochondrial membranes [31, 32], affecting their susceptibility to peroxidation by ROS [33]. Oxidative stress is recognized as an important trigger in the development of cardiovascular disease [34]. Mitochondrial β-oxidation of fatty acids is the major source of energy for the heart. Mitochondria are also central to stress-induced programmed cell death. In addition, in non-phagocytic cells, these organelles are the principal site of ROS production, via the electron transport chain [35]. Our results showed that palmitic acid-induced ROS generation through mitochondria not from NADPH complex (Fig. 1e, f). Antioxidant defense enzymes include superoxide dismutase (SOD), glutathione peroxidase (GPx) and catalase.

There are three types of SOD expressed in blood vessel: cytosolic Cu/Zn-SOD, Mn-SOD localized in mitochondria, and an extracellular form of Cu/Zn-SOD [36]. SOD protects against superoxide-mediated cytotoxicity by rapidly dismutating O_2_– to H2O2. Cu/Zn-SOD, after treated with palmitic acid, decreased Cu/Zn- SOD and Mn-SOD protein expression level (Fig. 1g). Palmate-induced apoptosis in the neonatal cardiomyocyte is associated with a decrease in the mitochondrial membrane potential, also decreasing the ability of the mitochondria to produce ROS [35]. ROS have been implicated in signal transduction pathways leading to a modulation of the DNA-binding activities of the transcription factor NFκB [37], implying a role for alterations in gene transcription as a response to oxidative stress, p38 MAPK, NH2-terminal Jun kinases/stress-activated protein kinases (JNK/SAPK), advanced glycosylation end-products (AGE)/receptor for AGE (RAGE), and protein kinase C (PKC) [38].Our results showed that palmitic acid induced ROS generation and decreased mitochondria membrane potential, also downregulated the antiapoptotic (Bcl2 and p-AKTser473) and upregulated the proapoptotic (Bax) proteins, furthermore, increased caspase 3 activity in H9c2 cells (Fig. 2b). Palmitic acid disruption of mitochondrial membrane function results in the discharge of the mitochondrial enzyme cytochrome c into the cytosol. In our study, we found that palmitic acid increased JNK/MAPK protein expression, but not p38 MAPK and ERK (Fig. 2d).We assumed that palmitic acid induced-damage through JNK/SAPK dependent pathway. The stress-activated protein kinases JNK1 and IKKβare central signal transducers in innate immunity and stress responses that control the expression of several proinflammatory genes [39]. Recently it has become evident that interference with either JNK1 or IKK activity improves insulin signaling in mouse models of obesity and lipid-induced glucose intolerance [40, 41]. Moreover, JNK and IKKβare also downstream of pathways activated by toxic lipids and excessive glucose levels [39]. In addition to JNK activation [42], oxidative stress was proposed to activate NFκB [43–45]. However, the link between NFκB and ROS has become complex because NFκB activation has anti-oxidant functions [42, 46, 47]. JNK activation may, however, promote ROS accumulation [48] and link ROS production to insulin resistance and loss of β-cell function [49–51]. We therefore used siRNA to figure out whether JNK/ NFκB pathway could mediate cardiomyocyte cell apoptosis induced by palmitic acid. As shown in Fig. 2g, NFκB has no influence on JNK activation.

**Fig. 5:**
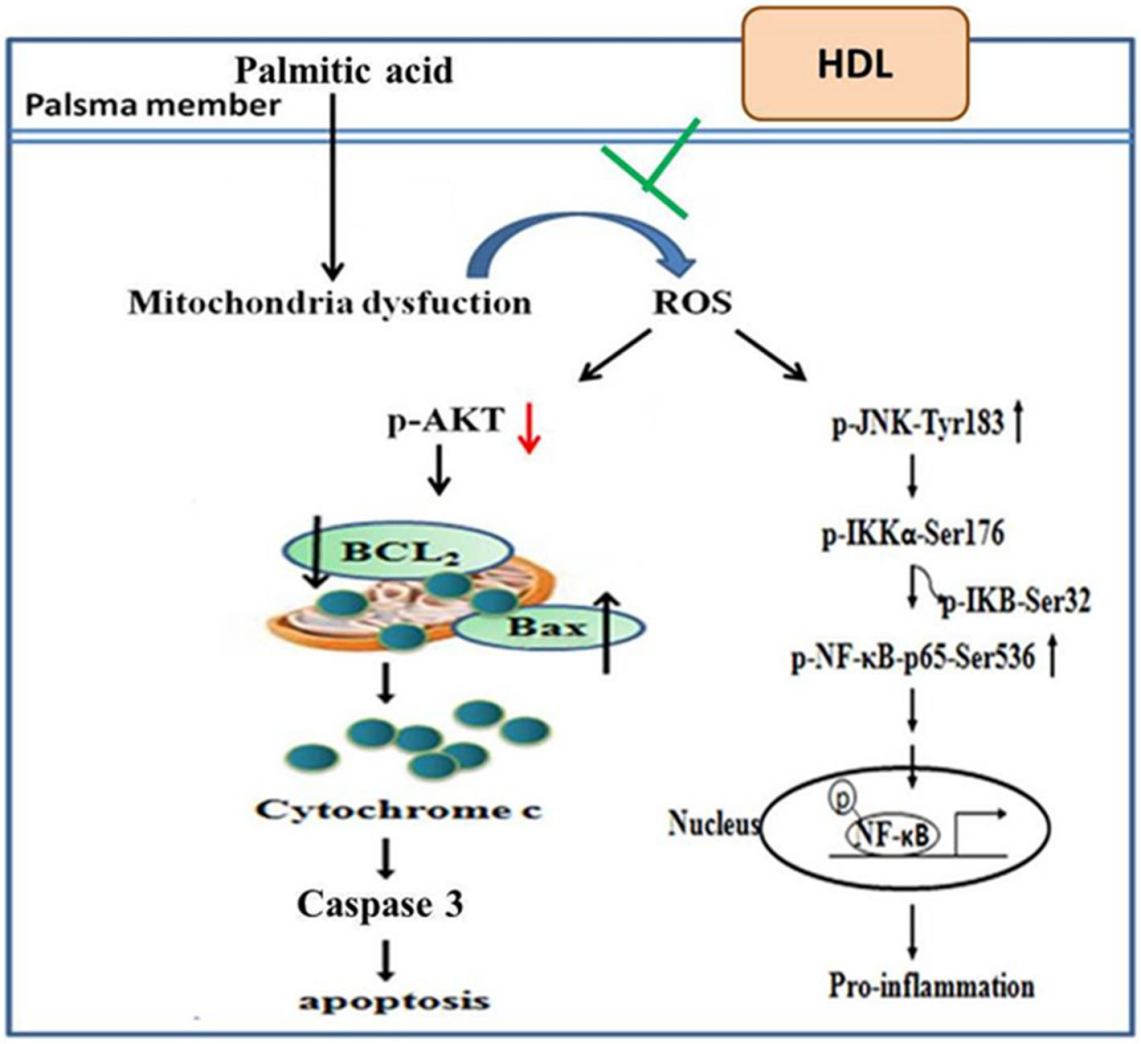
The proposed mechanism of high-density lipoprotein ameliorates palmitic acid-induced lipotoxicity and oxidative dysfunction

Epidemiological and clinical studies have demonstrated the inverse association between HDL cholesterol levels (HDL-C) and the risk of coronary heart disease (CHD) [21, 52]. Low HDL-C is the most frequent dyslipoproteinemia in patients with premature infarction [53] and is an independent predictor of recurrent coronary events [54, 55]. Furthermore raising HDL, decreases the incidence of coronary artery disease [56]. Several different actions are attributed to HDLs, which taken all together, have an anti-atherogenic effect. The primary action is the reverse cholesterol transport, mentioned above. Other actions have been described in vitro and in animals, such as: antioxidant, anti-inflammatory, platelet antiaggregant, anticoagulant, profibrinolytic, and endothelial protection effects [57–59]. In summary, the present results indicated that HDL attenuates palmitic acid-induced cardiomyocyte lipotoxicity and oxidative dysfunction via modulating mitochondria dependent pathway and p-JNK/NFκB signaling (Fig. 5). Therefore, reduce the downstream of superoxide-induced ROS generation and impairment of antioxidant enzymes, and inflammatory protein expression (Fig. 4c). In addition, HDL inhibited palmitic acid-induced cell death and apoptosis in cardiomyocytes (Fig. 4b). Further studies are required to confirm the effect of HDL on the inhibition of palmitic acid mediated pro-atherogenic effects and the effectiveness in vivo. Our findings may be a relevant therapeutic molecular mechanism in the improvement of cardiovascular disease. In H9c2 cells, several lines of evidence demonstrated that palmitic acid are taken up by the heart either via CD36/FATP transporters [60]. Whether HDL protects the cells against palmitic acid-induced apoptosis via Apo A-1, SR-B1 or other receptors will be another issue we can identify in the future study.

## Conclusion

Our results showed that PA-induced ROS accumulation which result in cardiac apoptosis and inflammation. However, HDL reduced PA-induced lipotoxicity and oxidative dysfunction via ROS suppression (Fig. 5). These results may provide insight into a possible molecular mechanism underlying HDL suppression of palmitic acid-induced cardiomyocyte apoptosis.

## Abbreviations

HDL: High-density lipoprotein
NEFA: Non-esterified fatty acid
PA: Palmitic acid
ROS: Reactive oxygen species
